# Augmentation of [^18^F]-C-SNAT4 PET imaging of apoptosis after radiotherapy using a cold mixing strategy

**DOI:** 10.1186/s13550-025-01255-1

**Published:** 2025-06-03

**Authors:** Jeffrey Qiu, Min Chen, Zixin Chen, Corinne Beinat, Stavros Melemenidis, Edward Graves, Jianghong Rao

**Affiliations:** 1 Department of Radiology, Molecular Imaging Program at Stanford, Stanford University, Stanford, CA 94305 USA; 2 Department of Radiation Oncology, Stanford University, Stanford, CA 94305 USA

**Keywords:** Apoptosis, Tumor therapy response, Preclinical PET imaging, Radiotherapy, Caspase-3

## Abstract

**Background:**

Positron Emission Tomography (PET) imaging can monitor cancer treatment response by non-invasively detecting apoptosis in vivo. Signal-to-noise (SNR) remains one of the critical barriers to approval for clinical use. We have previously developed a PET tracer [18 F]-C-SNAT4 for imaging capase-3 activity in apoptotic tumors induced by chemo- and immunotherapy. [18 F]-C-SNAT4 is designed to undergo caspase-3 activated intramolecular cyclization. The product then self-assembles in situ into nanoparticles to generate preferential retention of F18 radioactivity in apoptotic cells. This unique mechanism prompted us to investigate if a cold mixture could enhance the probe retention and further augment the sensitivity for imaging radiotherapy.

**Results:**

[18 F]-C-SNAT4 and hot/cold mixture [18 F]/[19 F]-C-SNAT4 were used to detect human NSCLC (NCI-H460) apoptosis induced by radiation. Both hot [18 F]-C-SNAT4 and hot/cold mixture [18 F]/[19 F]-C-SNAT4 had significantly increased uptake in radiation treated vs. untreated NCI-H460 cells in vitro. A 1: 80 hot/cold mixture increased signal by 1.6x compared to [18 F]-C-SNAT4 alone. In vivo studies were performed in murine xenograft models in high-dose radiation and low-dose radiation treatment groups. The hot/cold mixture showed an increase in the signal by 2.5x in high-dose radiation treated murine NCI-H460 xenograft models. Low-dose radiation induced apoptosis was only detected with the hot/cold mixture with 2.4x signal compared to hot [18 F]-C-SNAT4. Toxicity and dosimetry safety were evaluated at 250x and 10x respective dosages, then normalized to human dose equivalent.

**Conclusion:**

A hot/cold mixture of [18 F]/[19 F]-C-SNAT4 generates significantly more signal compared to hot [18 F]-C-SNAT4, leading to higher sensitivity in detecting treatment response. This may present a solution to low sensitivity in the translation of apoptosis-specific radionuclides to clinical application.

## Introduction

Methods to rapidly detect cancer treatment response remain elusive. The diversity of tumor biology in various malignancies focuses research attention on shared cellular pathways. Apoptosis is the central mechanism that unites the diverse armamentarium of current cancer therapy, from cytotoxic (chemotherapy, radiotherapy) to receptor-mediated modalities (immunotherapy, endocrine/targeted) [1–5]. Early detection of treatment-induced apoptosis in tumor cells holds the promise of personalized cancer medicine, where individual treatment response guides customized therapeutic regimens. Despite many advances in chemotherapy, its efficacy in solid tumors can be as low as 20–30%. Early recognition of treatment failure and initiation of alternative regimens can be lifesaving [6–8]. Current paradigms of treatment response rely heavily on cross-sectional imaging, with standardized guidelines in Response Evaluation Criteria in Solid Tumors (RECIST) 1.1 and Positron Emission Tomography Response Criteria In Solid Tumors (PERCIST) recommending imaging 4 weeks after treatment initiation [9, 10]. While waiting for repeat imaging, the cancer may continue to progress with ineffective therapy. For 4 weeks, the patient may be clinically worsening, while suffering adverse effects and diminished quality of life. Additionally, computed tomography (CT) and magnetic resonance imaging (MRI) are limited in sensitivity and specificity. These modalities have difficulty differentiating tumors from treatment response, particularly cellular processes of cytostasis, fibrosis, necrosis, or cystic change [11–12]. PERCIST was designed to address these shortcomings in the assessment of tumor response with cross-sectional imaging alone. However, traditional positron emission tomography (PET) using ^18^F-fluorodeoxyglucose (FDG) has several limitations due to the non-specific nature of glucose metabolism. Treatment-induced hypoxia and inflammation both increase FDG standardized uptake values (SUV) in the tumor microenvironment (TME), leading to the unreliable correlation of SUV to true histopathologic tumor response [13–16].

Radionuclide probes in development have the potential to shorten treatment response detection time from months to days, as apoptosis occurs within 48 h of initiation in all current cancer treatment modalities. Conversely, the absence of apoptosis in tumor cells is one of the most reliable predictors of treatment resistance [17–18]. By leveraging this universal mechanism of cancer cell death, a rapid and generalizable biomarker of treatment response can be realized [19–21]. The key mediators of apoptosis remain targets of intense research focus in treatment response imaging. In both extrinsic and intrinsic apoptotic signaling cascades, caspase-3 activation represents the final committed step of programmed cell death. Once activated by caspase-8 (extrinsic) or caspase-9 (intrinsic), this central scavenging cysteine protease cleaves the peptide sequence DEVD-X (DEVD Asp-Glu-Val-Asp, X = any amino acid) between D and X. This combination of specificity to irreversibly apoptotic cells and a well-defined “trigger mechanism” in the DEVD-X peptide sequence makes caspase-3 an ideal target as specific biomarkers of apoptosis [22–24].

One strategy to create caspase-3 specific radiotracer probes utilizes known inhibitors of caspase-3. These small molecules are pharmacokinetically advantageous, and form enzyme-inhibitor complexes for intracellular signal retention. Within this group, the isatin-5-sulfonamide derived [^18^F]-ICMT-11, has been most extensively validated in pre-clinical studies. In murine models, it predicted treatment response in allograft and xenograft models of malignancy, including breast, non-small cell lung cancer (NSCLC), and B-cell lymphoma. [^18^F]-ICMT-11 also demonstrated remarkable specificity in negative studies on caspase-3 knockout and necrosis models [24–29]. In the Phase 2 trial against breast carcinoma and NSCLC, [^18^F]-ICMT-11 failed to demonstrate post-treatment changes in PET tumor signal, despite increased M30/M65 ratio in cytokeratin-18 analysis - a highly sensitive biomarker for caspase-3/7 activity. The absolute signal intensity of caspase-3 inhibitor probes may be limited by the saturation of active binding sites [30].

Another strategy to formulate caspase-3-specific biomarkers utilizes its proteolytic activity to increase signal amplification within apoptotic cells. DEVD (Asp-Glu-Val-Asp) is the caspase-3 specific peptide sequence to activate tracer molecules. [^18^F]-CP18 uses the DEVD peptide sequence to allow caspase-3 to remove its C-terminus polyethylene glycol (PEG) chain, trapping the highly polar molecule within the apoptotic cytoplasm [31–32]. In preclinical murine models, [^18^F]-CP18 demonstrated caspase-3-dependent enrichment in glioblastoma and colon cancer xenografts [33–34]. Phase 2 trial of [^18^F]-CP18 to visualize treatment response to Birinapant in refractory epithelial ovarian/fallopian tube cancer is in progress (NCT01766622) [35].

**Scheme 1:**
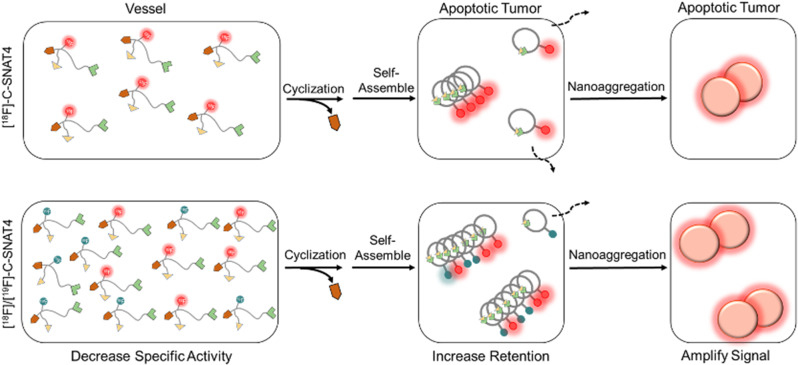
Schematic mechanism of increasing the sensitivity via [^18^F]-C-SNAT4 mixing with [^19^F]-C-SNAT4

Radiotracers derived from small molecules are attractive candidates due to rapid penetration of tumor cell membranes, however, they are also prone to washing out. Nanoaggregating probes harness the pharmacokinetic advantages of small molecules, but when activated, they self-assemble into large hydrophobic nanoparticles. The first such probe is described in our previous work on [^18^F]-C-SNAT, which undergoes caspase-3 activated intramolecular cyclization [22]. The end product is structurally rigid and hydrophobic, self-assembling in situ into nanoparticles via pi-pi stacking interactions (Scheme 1). Both large size and hydrophobicity present barriers to cellular clearance, promoting high signal retention [22]. [^99m^Tc]-AnnexinV and [^18^F]-ML-10, which target apoptosis-associated cell membrane changes, were among the most extensively studied in the pre-clinical literature. In vivo results showed that [^18^F]-C-SNAT was best in predicting treatment response [36]. A 2nd generation probe, [^18^F]-C-SNAT4 improves performance in serum stability, as well as increased signal-to-noise ratio (SNR) as measured by tumor/muscle uptake ratio (T/M) [37]. This study reports methods to improve SNR in our improved probe [^18^F]-C-SNAT4. By injecting a mixture of hot [^18^F]-C-SNAT4 and cold [^19^F]-C-SNAT4, we can increase the formation of intracellular nanoaggregates and improve sensitivity in detecting apoptotic cells (Scheme 1). We validated this methodology against xenografted NCI-H460 NSCLC in murine models treated with radiotherapy.

## Materials and methods

### Study design

The objective of this study was to further enhance the performance of [^18^F]-C-SNAT4 as an apoptosis imaging probe in radiotherapy. Utilizing a hot/cold mixing strategy, we aim to improve cellular retention of [^18^F]-C-SNAT4 within apoptotic cells. Adding 80x to 200x cold [^19^F]-C-SNAT4 substrate for caspase-3 facilitates the formation of intracellular nanoaggregates, which increases the local concentration of [^18^F] within apoptotic tumor cells. This study aims to quantify the increase in PET signal resulting from this cold mixing strategy in both in vitro and in vivo models. To adequately power the study at 90% and 5% significance level, 3–5 animals per group were used for the evaluation of [^18^F]-C-SNAT4 in xenograft tumor models in vivo, as well as in vitro studies. All experiments were performed in accordance with the National Institutes of Health Guide for the Care and Use of Laboratory Animals, as well as the ARRIVE (Animal Research: Reporting of In Vivo Experiments) guidelines. These protocols were approved by the Stanford University Institutional Animal Care and Use Committee.

### Cell line maintenance

Human NSCLC cell line NCI-H460 (ATCC) used for xenografts was regularly assessed for contamination and pathogens. NCI-H460 cells were cultured in Dulbecco’s modified Eagle’s medium (DMEM) (Invitrogen), supplemented with 10% v/v fetal bovine serum (Life Technologies Inc.) and 1% v/v penicillin-streptomycin (100 IU/ml, 100 mg/ml; Life Technologies Inc.). Incubation conditions were set at 37 °C, humidified, and 5% CO_2_. Irradiation protocol was set at room temperature with a dose rate of 0.2 Gy/min (0.2 mm Cu and 1 mm Al filter, 150 kV, Hitachi). Post-treatment viability was assessed with CellTiter96^®^ Aqueous One Solution Cell Proliferation Assay (MTS) kit from Promega (Madison, WI, USA), following the recommended protocol.

### Probe synthesis

The synthesis of [^18^F]-CSNAT followed our previously reported procedure [37, 38]. Briefly, ^18^F-azide was synthesized via a fully automated TRACERlab FX-FN module (GE Healthcare, USA) and purified by semi-preparative high-performance liquid chromatography (HPLC) before its use for conjugation to the precursor C-SNAT4 by a copper-catalyzed azide-alkyne cycloaddition (CuAAC) in the presence of an accelerating ligand (BimC4A)_3_. The final product [^18^F]-C-SNAT4 was purified by semi-preparative HPLC and formulated in saline with 10% ethanol by solid phase extraction with a C-18 Sep-Pak light cartridge.

### Radiotracer uptake study

[^18^F]-C-SNAT4 uptake analysis in NCI-H460 cells (2 × 10^5^) started with overnight culture in 6-well cell culture plates. Radiotherapy treatment cells were irradiated with 10 Gy using a single 225 kV beam Kimtron IC225 irradiator (Kimtron Medical, CT, USA). 24 h post-treatment, 1.48 MBq (40 µCi) [^18^F]-C-SNAT4 in RPMI medium 1640 was added (1 ml/well; 13.3 pmol). At intervals of 10, 30, and 60 min after the addition of [^18^F]-C-SNAT4, the supernatant was aspirated. Cells were lifted with trypsin, then washed in cold PBS 3 times. The radioactivity level of each cell pellet was measured with a gamma counter and then normalized to total protein concentration. The protein content was quantified by the Bradford method after cell digestion using RIPA buffer. [^18^F]-C-SNAT4 uptake concentration was calculated in µM of % uptake/mg protein. Results were normalized to the untreated control cells following the same protocol.

### Animal model and treatment

For xenografting, NCI-H460 (2 × 10^6^ cells in 100 µl of PBS) cells were injected subcutaneously on the back of female nu/nu nude mice (aged 6 to 8 weeks; Charles River Laboratories). Tumor dimensions were measured with a caliper (by the same researcher). Calculated volume = a^2^ × b/2, where a and b represent the width and length of the tumor, respectively. Treatment-response analysis was performed 24–48 h post-treatment. In the radiotherapy mouse model, treatment consisted of two fractions of 5 Gy–2 Gy dose of radiation therapy delivered with a single 225 kV beam using the Kimtron IC225 irradiator (Kimtron Medical, USA).

### Small-animal PET/CT imaging and analysis

PET/CT imaging utilized a Siemens Inveon PET/CT scanner (128 × 128 × 159 matrix; CT attenuation and non-scatter corrected) (Siemens Medical Solutions USA). Intravenous injection of 200 µCi [^18^F]-C-SNAT4 was carried out by tail vein access in NCI-H460 xenografted mice (3–5 per group). Dynamic scanning was performed at 22 times frames over 90 min (list mode), and reconstructed at 0.5 sinogram bins (4 × 15s, 4 × 60s, 11 × 300s, 3 × 600s). Anesthesia protocol used 2% isoflurane. The tumor region was positioned at the center of FOV for optimal resolution. 3D ordered-subsets expectation maximization (3D-OSEM) utilized for iterative reconstruction with fast maximum a posteriori (fast MAP) set at MAP OSEM iterations, 2; MAP subsets, 16; MAP iterations, 18. Volume rendered images (VRT) to visualize [^18^F]-C-SNAT4 uptake were produced by Siemens Inveon Research Workplace software v.4.0 after manual setting of volumes of interest (VOI). Relative radioactivity was calculated by mean pixel values of multiple ROI volumes, converted to counts/mL/min, then normalized to injected dose (ID) to obtain percent ID per cubic centimeter of tissue (%ID/cc).

### Ex vivo immunofluorescence

Slides for microscopy prepared in 5-µm thick slices of formalin-fixed tumors in paraffin following manufacturer protocol (Histo-Tec Laboratory). Section samples representative of the entire tumor volume were fixed in formalin for 10 min, washed, permeabilized with 0.5% Triton X-100 in PBS, and blocked with 3% BSA and 3% goat serum. Primary staining was performed with rabbit-derived anti-cleaved caspase-3 (Cell Signaling Technology Inc., MA, USA) incubated overnight at 4 °C, then incubated in Alexa 647-goat-anti-rabbit IgG secondary antibody (Life Technologies) and Alexa 488-phalloidin conjugate (Life Technologies) for 1 h at room temperature, covered to prevent photobleaching. Slides were mounted in SlowFade with DAPI (Life Technologies), sealed, and imaged on AxioObsever.Z1 confocal microscope with LSM 710 laser scanning module (Carl Zeiss AG, Ltd.). Immunofluorescence imaging was acquired at 405 nm (DAPI), 488 nm (Alexa 488), and 633 nm (Alexa 647), using 20 × (Plan Apochromat, NA = 0.8) and 63 × oil immersion (NA = 0.19) objectives.

### Statistics

Statistical calculations used GraphPad Prism v.8 (GraphPad Software Inc., CA) and expressed in means ± standard deviation (SD). One-way ANOVA with post-hoc Tukey test was used in the two-group analysis. General linear model repeated-measures analysis used for group time course analysis. One-tailed Pearson’s *r* was used to perform correlation analyses. *P* < 0.05 was used as a cut-off for statistical significance as indicated in the figures.

## Results

### Mixture strategy for monitoring response to radiation in cells

The process of [^18^F]-C-SNAT4 nanoaggregation involves non-covalent intermolecular interactions of macrocyclics derived from the activation of the tracer by caspase-3, which is concentration-dependent. When little [^18^F]-C-SNAT4 is available to be cleaved and turned into macrocyclic products, there will be less ^18^F activity retained in tumor cells. The uncleaved tracer will diffuse back into circulation for rapid renal clearance (Scheme 1). Based on the unique in-situ aggregation mechanism of [^18^F]-C-SNAT4, if we can mix the radiotracer with cold [^19^F]-C-SNAT4, we may increase the overall concentration of macrocyclic products (mixture containing [^18^F] and [^19^F] labels) in treated tumors and finally promote the formation of more aggregates at the target site. Therefore, we sought to examine whether mixing with cold [^19^F]-C-SNAT4 could improve the sensitivity of [^18^F]-C-SNAT4.

**Fig. 1:**
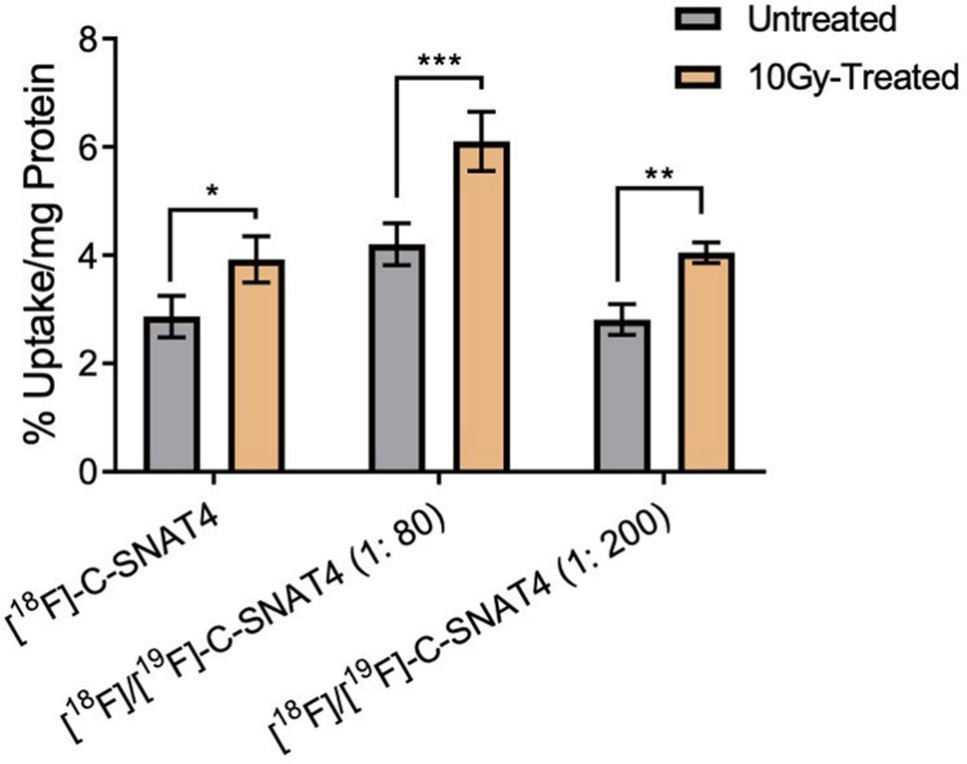
Mixture tracers of [^18^F]/[^19^F]-C-SNAT4 enhance uptake in radiation (a single dose at 10 Gy) treated cells and untreated NCI-H460 cells. 40 µCi of [^18^F]-C-SNAT4 or mixed with [^19^F]-C-SNAT4 (at a ratio of 1:80 or 1:200) were incubated in cells for 60 min at 24 h post-irradiated treatment and radioactivity of ^18^F was measured using ɤ-counter. Data are means ± s.d., ****P < 0.0001 (ANOVA), *n* = 3 for each group

One caveat of this strategy is that the addition of cold [^19^F]-C-SNAT4 leads to an increased number of substrates that will compete for the enzyme active site. We expect both the cold and hot C-SNAT4 to possess the same *kcat* and Kd for caspase-3, and the addition of cold [^19^F]-C-SNAT4 can result in the reduction of activated [^18^F]-C-SNAT4 and less cyclized hot product. Therefore, we first optimized the mixing ratio of [^18^F]/[^19^F]-C-SNAT4 to balance aggregation and enzyme activation, providing the best signal retention. Two different doses of cold [^19^F]-C-SNAT4 (0.9 nmol and 2.4 nmol) were added to 40 µCi of hot [^18^F]-C-SNAT4 prior to ionizing radiation treatment to induce apoptosis (Fig. 1). And the respective mixing ratios of [^18^F]/[^19^F]-C-SNAT4 were calculated at 1: 80 and 1: 200 based on the specific activity of [^18^F]-C-SNAT4 (3.4 Ci/µmol). At 24 h after 10 Gy radiation, the mixed hot/cold tracer [^18^F]/[^19^F]-C-SNAT4 or unmixed tracer [^18^F]-C-SNAT4 were both incubated in irradiated NCI-H460 cells for up to 1 h. In irradiated cells, the group treated with [^18^F]/[^19^F]-C-SNAT4 (1:80) showed 6.1 ± 0.5% uptake/mg protein, showing 1.6-fold increase from the tracer [^18^F]-C-SNAT4 (3.9 ± 0.4% uptake/mg protein) (*P* < 0.0001; *n* = 3). This result supported our hypothesis that mixing the cold analogue with the radioactive tracer could improve self-aggregation and signal retention. For mixture tracer [^18^F]/[^19^F]-C-SNAT4 at the ratio of 1: 200, the tracer uptake was 4.0 ± 0.2% uptake/mg protein, which has no significant increase compared to the unmixed group. The higher uptake of the mixture ratio at 1: 80 compared to the mixture ratio at 1: 200 may be due to the competition for the enzyme active site by the high amount of the cold analogue, which inhibited the activation of the radiotracer. For untreated cells, there was no significant difference among the three groups (2.9 ± 0.4% uptake/mg protein for the mixture ratio at 1: 80, 3.5 ± 0.2% uptake/mg protein for the mixture ratio at 1: 200 or 2.8 ± 0.3% uptake/mg protein for hot tracer only, respectively) (*P* > 0.05; *n* = 3). These results revealed that the mixture strategy can increase the cellular retention of ^18^F activity and improve the detection sensitivity in radiation-treated cells but not increase the cellular uptake in untreated cells.

### Mixture strategy for imaging response to radiation in vivo

**Fig. 2:**
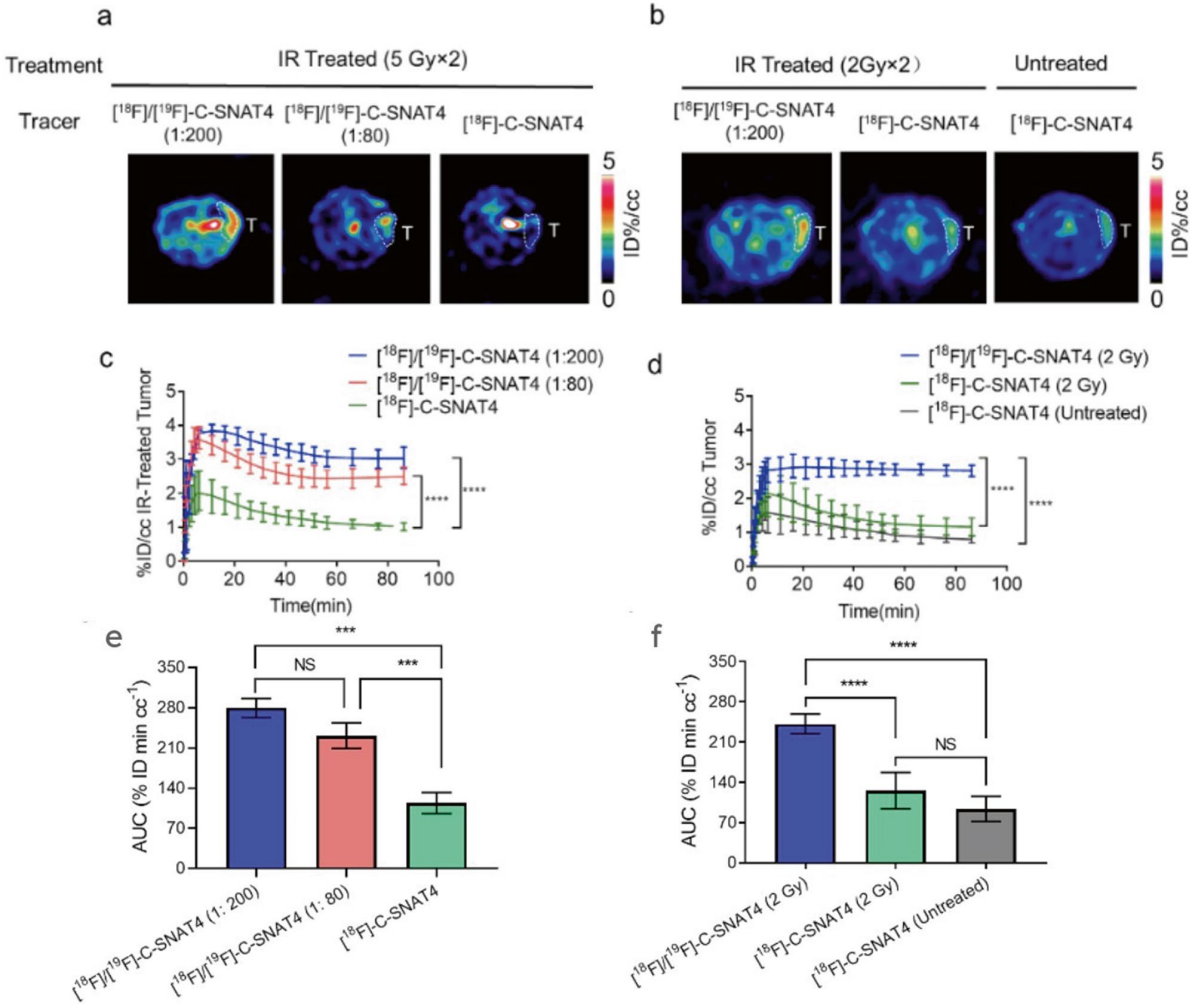
Increased sensitivity to radiotherapy treatment response using mixed tracers of [^18^F]/[^19^F]-C-SNAT4. (**a**) PET images of radiotherapy in NCI-H460 tumor model under irradiation therapy (5 Gy, 2 times). (**b**) PET images of radiotherapy in NCI-H460 tumor model under irradiation therapy (2 Gy x 2). Representative PET images of NCI-H460 tumor-bearing mice imaged following 24 h irradiation treatment at 1 h post-injection of approximately 200 µCi tracer intravenously. (**c**, **d**) Time-activity curve of 5 Gy (c) or 2 Gy (d) is shown from t = 0 to t = 85 min for TAC. For all TAC points represent mean ± s.d. (*n* = 3–4 for each group). (**e**) The area under the curve (AUC) was showed from t = 0 to t = 85 min for time activity of mixture tracer [^18^F]/[^19^F]-C-SNAT4 (1:200 Blue; 1:80 Red) and hot tracer [^18^F]/[^19^F]-C-SNAT4 (Green) in two fractions of 5 Gy-treated tumors (10 Gy total). (**f**) The area under the curve (AUC) was showed from t = 0 to t = 85 min for time activity of mixture tracer [^18^F]/[^19^F]-C-SNAT4 (1: 200, Blue) and hot tracer [^18^F]-C-SNAT4 (Green) in two fractions of 2 Gy-treated tumors and hot tracer [^18^F]-C-SNAT4 (Grey) in untreated tumors. Data are means ± s.d., ***P < 0.001; ****P < 0.0001 (ANOVA), NS = not statistically significant, *n* = 4 each group

We next evaluated this mixture strategy for monitoring radiotherapy outcomes in vivo. Subcutaneous NCI-H460 tumor was implanted in nu/nu mice, and when the tumor sizes reached approximately 150 mm^3^, they were treated with two fractions of 5 Gy (10 Gy total) radiation. At 24 h post-radiotherapy, mice were injected with mixture tracer [^18^F]/[^19^F]-C-SNAT4 (at a ratio of 1:80 or 1:200) or hot tracer only. Then the tumor uptakes of the three groups were evaluated by PET/CT imaging (Fig. 2a). As shown in the time activity curve (TAC), both groups injected with mixtures of tracer show higher uptake (3.0 ± 0.3% ID/cc for mixture ratio at 1:200 and 2.5 ± 0.3% ID/cc for mixture ratio at 1:80) than the group injected with only hot tracer (1.0 ± 0.3% ID/cc) at 85 min post-injection (Fig. 2c). The area under the curve of TAC from t = 0 to t = 85 min (Fig. 2e) showed that the mixture groups (279.4 ± 16.6% ID min/cc for 1:200 ratio; 230.9 ± 22.4% ID min/cc for 1:80 ratio) had 2.5x increase in uptake compared to hot tracer alone (113.9 ± 18.4% ID min/cc) (*P* < 0.0001; *n* = 4). Different from what we observed in the cell uptake experiment, there is no significant difference in uptake between the two mixture groups in vivo. It may be due to the complex interplay of the tumor microenvironment, tracer pharmacokinetics, and heterogeneous tumor response to radiotherapy, in addition to a high level of caspase-3 that can efficiently process both cold and hot tracers.

**Fig. 3:**
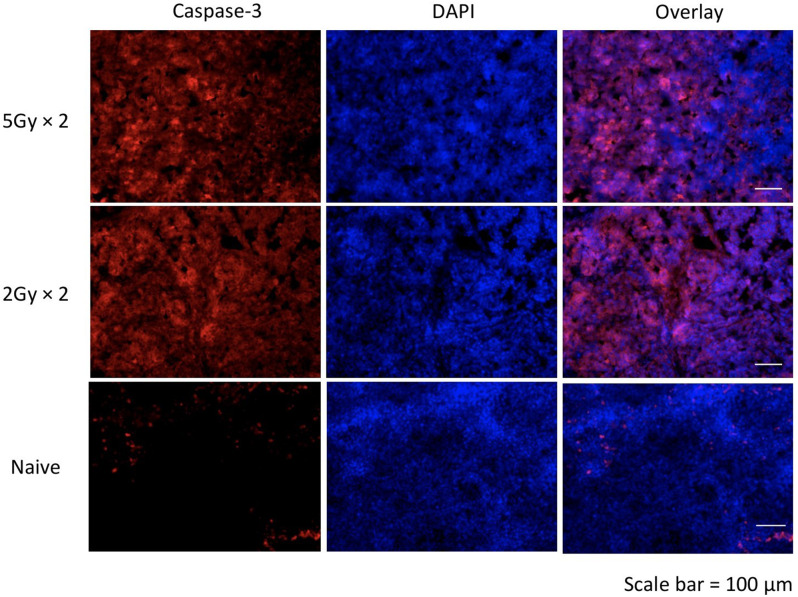
Ex vivo immunofluorescence of tumor sections. Negative control (treatment naïve) tumor slides show minimal caspase-3 activity. Tumors treated with high dose (5 Gy x 2) vs. low dose (2 Gy x 2) radiotherapy demonstrate effective induction of caspase-3 mediated apoptosis under radiotherapy conditions

The finding that the mixture strategy could improve the imaging contrast in the 5 Gy-treated tumor model led us to validate this strategy in low-dose radiation treatment, which could induce low levels of caspase-3. We then used mixture tracer [^18^F]/[^19^F]-C-SNAT4 (1: 200) to image the treatment efficiency in a low-dose treatment using two fractions of 2 Gy-treatment (4 Gy in total) for tumor-bearing mice (Fig. 2b). The TAC data (Fig. 2d) showed tracer uptake was significantly enhanced by 2.4-fold in tumor following injection of [^18^F]/[^19^F]-C-SNAT4 tracer at the ratio of 1: 200 (2.8 ± 0.2% ID/cc) compared to [^18^F]-C-SNAT4 only (1.2 ± 0.3% ID/cc) in 2 Gy-treated tumor-bearing mice. Using [^18^F]-C-SNAT4 for PET imaging could not differentiate the treated tumor (1.2 ± 0.3% ID/cc) from the untreated tumor (0.8 ± 0.1% ID/cc) (*P* > 0.05; *n* = 4). The AUC value of mixture tracer [^18^F]/[^19^F]-C-SNAT4 (ratio at 1: 200) uptake in treated tumor (Fig. 2f) was 242.0 ± 17.2% ID min/cc, which was higher than the AUC value of [^18^F]-C-SNAT4 in treated (123.6 ± 30.9% ID min/cc) and untreated tumors (92.1 ± 20.9% ID min/cc) (*P* < 0.0001; *n* = 4). Radiotherapy treatment conditions were validated with ex vivo immunofluorescence microscopy and caspase-3 staining provided by Alexa 647-goat-anti-rabbit IgG. Both high-dose (5 Gy x 2) and low-dose (2 Gy x 2) treatments produced a robust and uniform caspase-3 dependent apoptosis response (Fig. 3), supporting 10 Gy and 4 Gy doses as treatment conditions in the detection of caspase-3 activity. These in vitro and in vivo data indicated that the mixture strategy could enhance ^18^F activity retention and improve sensitivity for monitoring the therapeutic efficacy after low-dose radiation treatment.

### Dosimetry study of for clinical translation

The promising mechanistic and biological profiles of [^18^F]-C-SNAT4 support its translation into clinical settings. Therefore, we performed a safety and dosimetry study. The safety and toxicity of [^19^F]C-SNAT-4 was studied by an independent third party (Sobran Inc., Fairfax, VA). An experimental group of 10 male and 10 female Sprague-Dawley rats were intravenously dosed with 0.2775 mg/kg of [^19^F]C-SNAT-4. A control group of 10 male and 10 female rats was given a placebo of 10% ethanol in 0.9% sodium chloride and 2.225% DMSO. Half of each group was bled, euthanized, and necropsied at Day 3, and the other half at Day 15. All animals survived until euthanasia. There were no significant differences in clinical parameters such as body or organ weight. Clinical and gross pathologic analysis demonstrated no differences between groups. Laboratory parameters at Day 3 showed a statistically significant (*p* < 0.05) increase in alkaline phosphatase in male rats dosed with [^19^F]C-SNAT-4. At Day 15, female rats given [^19^F]C-SNAT-4 had significantly (*p* < 0.05) elevated hemoglobin, cholesterol, triglycerides, alkaline phosphatase, and total protein compared to the control group. Urinalysis and coagulation panel demonstrated no difference at any time point. The report concluded that 0.2775 mg/kg of [^19^F]C-SNAT-4 was well tolerated, without clinically significant abnormality in pathologic or laboratory analysis. This result suggested that the toxicity of this tracer was minimal. It also confirmed that the mixture ratio at 1: 200 (Table 1) is still within the safe range.

Then we estimated human absorbed radiation based on distribution data for normal mice. Four male and female nude mice received an intravenous injection of [^18^F]-C-SNAT4 (~ 400 µCi). We performed 60 min-dynamic PET/CT imaging and static PET/CT scan at 4 h post-injection to assess radiation dosimetry. Regions-of-interest (ROIs) were manually drawn on organs of interest (heart, liver, kidney, spleen, intestine, muscle, bone, and bladder). The activity concentrations in these organs [%ID/g] were used for the dosimetry calculation. Then the source organ residence times were exported to the OLINDA/EXM program for computation of the human-absorbed doses. As shown in Table 1, human estimates of [^18^F]-C-SNAT4 revealed that the spleen receives the highest dose in females (0.14 rem/mCi) and males (0.10 rem/mCi). As described the maximal radiation dose to an adult’s whole body in the Code of Federal Regulations 21CFR§ 361.1(b)(3)(i), active blood-forming organs, lens of the eye, and gonads is 3.

Rem/single dose (5 Rem/year) and 5 Rem/single dose (15 Rem/year) for the other organs. This translates to a maximum allowed [^18^F]-C-SNAT4 dose of 36.23 mCi per scan (108.70 mCi per year) for females and 52.25 mCi per scan (156.74 mCi per year) for males. When an adult patient was administered at a single dose of 10 mCi radioactivity for [^18^F]-C-SNAT4 in the clinic, the equivalent doses (ED) would be 1.38 Rem for females and 0.96 Rem for males. Therefore, the doses patients received at 10 mCi of [^18^F]-C-SNAT4 are well within the dose limits specified.

**Table 1:**
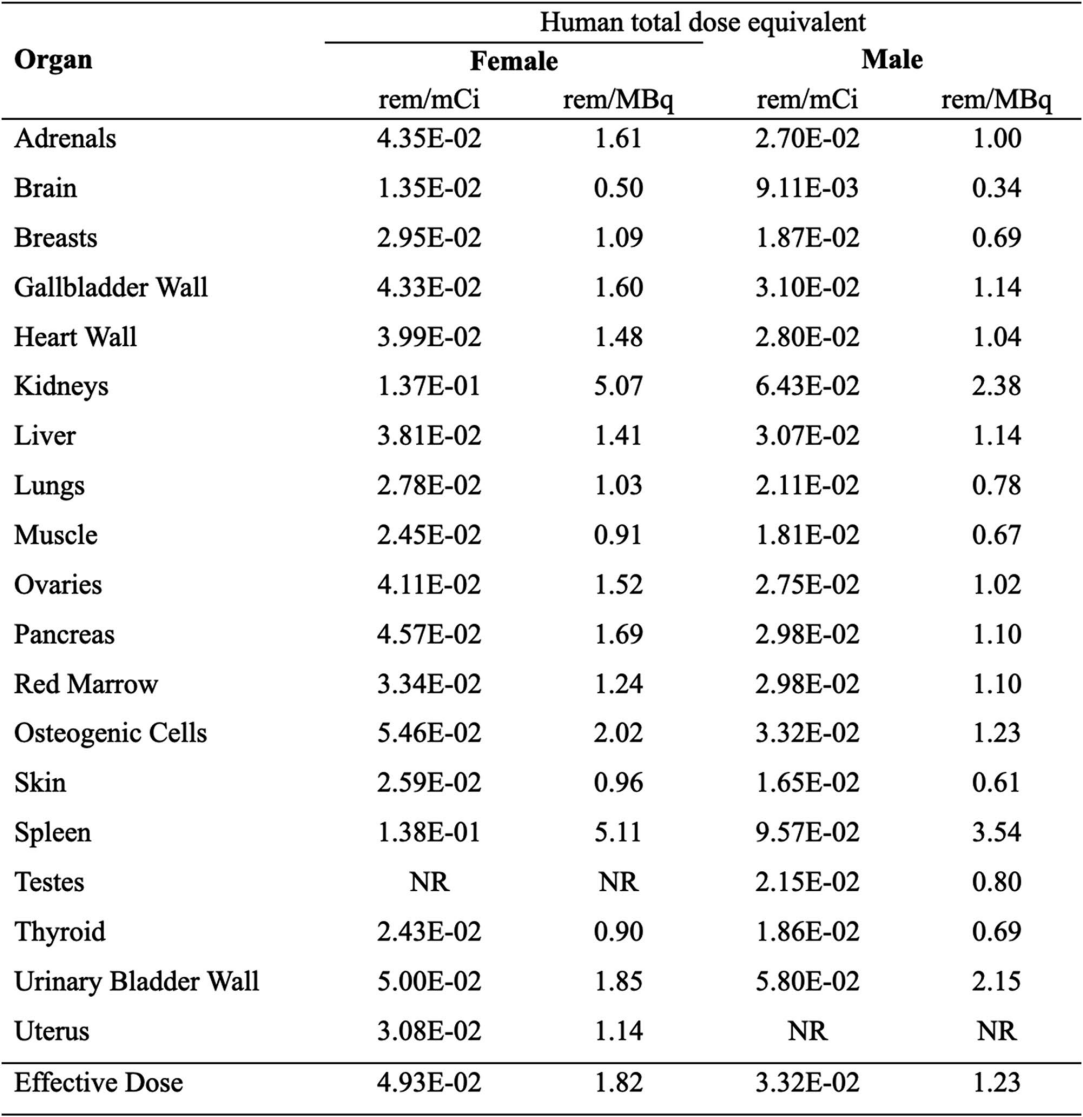
Estimated radiation dose to an adult female and male (human) after IV injection of [^18^F]-C-SNAT4.*

*The values are calculated using OLINDA software based on the micro-PET/CT imaging data obtained in NU/NU mice (female *n* = 3; male *n* = 3)

## Discussion

Chemotherapy response rates can range as low as 20% for first-line therapy. Thus, up to 80% of patients are suffering adverse effects of cytotoxic therapy for months, without any benefit [6]. High-affinity probes for apoptotic activity can predict treatment response in hours. Yet, as of this writing, no probe has been approved for clinical practice despite several candidates clearing Phase 1 trials for safety [29]. Among small molecular caspase-3 probes, which demonstrate favorable pharmacokinetics and tumor penetration properties, such as [^18^F]-ICMT-11 and [^18^F]-ML-10, SNR remains the primary barrier to clinical application [29–30, 36]. [^18^F]-C-SNAT4 generates significantly higher SNR in numerous preclinical studies [37–38]. A hot/cold mixing strategy to increase cellular retention can further multiply the signal output by 1.7–2.5x, directly addressing the most critical failure point of previous apoptosis probes in Phase 2 trials.

Specific radioactivity is a key determinant of sensitivity in molecular imaging. Binding sites, and reaction vs. elimination kinetics all limit the number of radiotracer-target interactions. Higher specific radioactivity allows for the detection of smaller concentrations of molecular targets by maximizing the signal output from a limited number of radiotracer-target-specific interactions [39–42]. The hot/cold ^18^F/^19^F mixing strategy developed here lowers the specific radioactivity, however, it has augmented the SNR of [^18^F]-C-SNAT4. Both 1: 80 and 1: 200 hot/cold mixtures increased tumor signal by 2.5x in the standard (2 treatments, 5 Gy each) radiotherapy model. The signal-boosting properties of a hot/cold probe mixture strategy have also been reported by Qiu et al. who rationally designed their [^18^F]-1 caspase-3 probe along similar principles to [^18^F]-C-SNAT4, with a DEVD recognition sequence, triggering intramolecular cyclization and nanoaggregation. A hot/cold mixture increased T/M by 1.3x in both intratumoral and intravenous administration of DOX in HeLa xenograft mouse models [43]. The case for [^18^F]-C-SNAT4 and [^18^F]-1, activity-based probes, is different according to the theorized mechanism in Scheme 1–higher substrate levels promote the formation of stable intracellular nanoaggregates, increasing the absolute number of [^18^F], and therefore PET signal. Given this mechanism, [^18^F]-C-SNAT4 benefits from a greater absolute increase in nanoaggregate formation with the hot/cold mixing strategy. On the other hand, first-order kinetics of intramolecular cyclization in [^18^F]-C-SNAT4 will see a greater absolute increase in reaction rate, compared to second-order dimerization required for [^18^F]-1 to form cytoplasmic nanoaggregates.

Radiotherapy alone has broad oncologic applications, yet treatment response imaging with apoptosis radiotracers has only previously been reported with [^99m^Tc]-Annexin-V and [^18^F]-ML-10. The Phase 2 SABR-COMET trial demonstrated a 240% increase in 5-year overall survival (42.3% vs. 17.7%) with the addition of radiotherapy in a broad range of oligometastatic malignancies [44]. In palliative applications, response rates for tumor bleeding, which are not amenable to any other treatments, exceed 80% [45]. Haas et al. deployed [^99m^Tc]-Annexin-V SPECT in 11 patients with Grade I/II follicular lymphoma, which suggested a correlation of pathologically complete response with SPECT signal. This was a qualitative study, limited by the lack of signal quantification and low resolution inherent to SPECT [46–47]. Treatment response of brain metastases to 30 Gy radiotherapy was quantified by voxel-based analysis using [^18^F]-ML-10, which demonstrated a clear correlation between % voxels with signal increase and % decrease in tumor volume. However, the study does not quantify absolute signal output, and cannot address previously stated limitations of low specific radioactivity and SNR of [^18^F]-ML-10 [48–49]. [^18^F]-C-SNAT4 with hot/cold mixing improves on these methodologies by providing quantitative signal, and exquisite sensitivity to radiation doses of just 4 Gy. This high sensitivity allows a hot/cold mixture of [18F]-C-SNAT4 a wide range of tolerances to unquantifiable variables in individual tumor biology, such as different expression levels of caspase-3/7.

To facilitate the translation of [^18^F]-C-SNAT4 to clinical application, we confirmed the safety of a 250x dose of [^19^F]-C-SNAT4, which would be higher than a 1:200 mixture. No toxicity or adverse effects were observed in rat models over 15 days. Given the small size of [^18^F]-C-SNAT4, the dominant elimination mechanism is renal, with rapid clearance observed 60 min after administration [37]. Dosimetry studies demonstrate safety at 10x the diagnostic dose in a human equivalent scan (0.1 Rem, annual limit of 5 Rem) [50], leaving a significant margin of safety to increase dosages to achieve sufficient signal for tissue penetration and diagnosis.

A small molecular probe for caspase-3/7 would not only be generalizable to any primary cancer but also detect the committed step of apoptotic tumor cell death with high specificity. These advantages are facilitated by bypassing significant challenges in sensitivity/specificity and pharmacokinetics of other treatment detection strategies [51–56]. In addition, the universality of apoptosis stands out against the overwhelming diversity of tumor biology and oncogenesis. All current cancer therapies produce significant, detectable apoptotic activity in tumors. Conversely, the absence of apoptotic activity remains one of the most reliable predictors of treatment non-response [17–21]. The detection of treatment response at 24 h demonstrated in this study could represent a paradigm shift in the current standard of care, in which detection times are measured in months.

## Conclusion

The high signal intensity of [^18^F]-C-SNAT4 in the detection of apoptotic tumor cells can be further augmented 2.5x by utilizing a hot/cold [^18^F]/[^19^F] probe mixture strategy to improve intra-tumoral signal retention. Rapid renal clearance allows for low background signal and minimal toxicity. [^18^F]/[^19^F]-C-SNAT4 mixture demonstrates significantly enhanced sensitivity to treatment response in low-dose radiotherapy, undetectable by [^18^F]-C-SNAT4 alone. Human clinical studies are needed to validate [^18^F]/[^19^F]-C-SNAT4 mixture in predicting treatment response and realize the dream of personalized cancer therapy.

## Data Availability

The datasets generated during and/or analysed during the current study are available from the corresponding author on reasonable request.
